# New multienzymatic complex formed between human cathepsin D and snake venom phospholipase A_2_

**DOI:** 10.1590/1678-9199-JVATITD-2022-0002

**Published:** 2022-11-04

**Authors:** Jeane do Nascimento Moraes, Aleff Ferreira Francisco, Leandro Moreira Dill, Rafaela Souza Diniz, Claudia Siqueira de Oliveira, Tainara Maiane Rodrigues da Silva, Cleópatra Alves da Silva Caldeira, Edailson de Alcântara Corrêa, Antônio Coutinho-Neto, Fernando Berton Zanchi, Marcos Roberto de Mattos Fontes, Andreimar Martins Soares, Leonardo de Azevedo Calderon

**Affiliations:** 1 Center for the Study of Biomolecules Applied to Health (CEBio), Oswaldo Cruz Foundation (FIOCRUZ), Porto Velho, RO, Brazil.; 2 Graduate Program in Experimental Biology (PGBIOEXP), Department of Medicine (DEPMED), Federal University of Rondônia (UNIR), Porto Velho, RO, Brazil.; 3 Department of Biophysics and Pharmacology, Botucatu Biosciences Institute (IBB), São Paulo State University (UNESP), Botucatu, SP, Brazil.; 4 Laboratory of Biotechnology of Proteins and Bioactive Compounds (LABIOPROT), Oswaldo Cruz Foundation (FIOCRUZ), Porto Velho, RO, Brazil.; 5 National Institute of Science and Technology of Epidemiology of the Western Amazon, INCT-EPIAMO, Porto Velho, RO, Brazil.; 6 Smart Active Ingredients Lab (SAIL), Porto Velho, RO, Brazil.; 7 Faculty of Medicine, University of Buenos Aires (UBA), Buenos Aires, Argentina.; 8 Federal Institute of Education, Science and Technology of Rondonia (IFRO), Porto Velho, RO, Brazil.; 9 Bioinformatics and Medicinal Chemistry Laboratory (LABIOQUIM), Oswaldo Cruz Foundation (FIOCRUZ), Porto Velho, RO, Brazil.; 10 São Lucas University Center (UniSL), Porto Velho, RO, Brazil.; 11 Aparicio Carvalho University Center (FIMCA), Porto Velho, RO, Brazil.

**Keywords:** Cathepsin D, Phospholipases A_2_, Snake venom, Enzyme complex

## Abstract

**Background:**

Cathepsin D (CatD) is a lysosomal proteolytic enzyme expressed in almost all tissues and organs. This protease is a multifunctional enzyme responsible for essential biological processes such as cell cycle regulation, differentiation, migration, tissue remodeling, neuronal growth, ovulation, and apoptosis. The overexpression and hypersecretion of CatD have been correlated with cancer aggressiveness and tumor progression, stimulating cancer cell proliferation, fibroblast growth, and angiogenesis. In addition, some studies report its participation in neurodegenerative diseases and inflammatory processes. In this regard, the search for new inhibitors from natural products could be an alternative against the harmful effects of this enzyme.

**Methods:**

An investigation was carried out to analyze CatD interaction with snake venom toxins in an attempt to find inhibitory molecules. Interestingly, human CatD shows the ability to bind strongly to snake venom phospholipases A_2_ (svPLA_2_), forming a stable muti-enzymatic complex that maintains the catalytic activity of both CatD and PLA_2_. In addition, this complex remains active even under exposure to the specific inhibitor pepstatin A. Furthermore, the complex formation between CatD and svPLA_2_ was evidenced by surface plasmon resonance (SPR), two-dimensional electrophoresis, enzymatic assays, and extensive molecular docking and dynamics techniques.

**Conclusion:**

The present study suggests the versatility of human CatD and svPLA_2_, showing that these enzymes can form a fully functional new enzymatic complex.

## Background

Cathepsins compose a family of lysosomal proteases mainly found in acidic endo/lysosomal compartments and are implicated in a broad spectrum of physiologic processes, such as intracellular protein degradation, energy metabolism, hormonal regulation, bone resorption, and immune responses [1]. These proteins belong to three protease families, characterized based on differences in the following amino acids at their active site: aspartic proteases (D and E), serine proteases (A and G), or cysteine proteases (B, C, H, F, K, L, O, S, V, X, and W) [1-4].

Furthermore, cathepsins are essential to maintaining cell homeostasis [5]. The inactivation, loss of function, and overexpression of these proteases can result in inappropriate degradation and abnormal accumulation of lysosomal waste [1, 6]. In addition, extracellular oversecretion of cathepsins is associated with uncontrolled cell proliferation, invasion, and differentiation, which in turn may bring about the onset of fatal pathologies, including atherosclerosis, cancer, and tissue fibrosis [6-12].

Due to its physio-pathological functions, cathepsin D (CatD) is one of the most studied lysosomal proteases [13-15]. CatD is an aspartic endopeptidase with two conserved Asp residues in its active site; these residues tend to deprotonate, indicating that the pH-optimum of activity resides at pH values below 5 [16]. In addition, CatD has three distinct regions that are characteristic of aspartic proteases, an N-terminal domain (residues 1-188), a C-terminal domain (residues 189-346), and an interdomain, antiparallel/3-sheet formed by the N-terminus (residues 1-7), the C-terminus (residues 330-346), as well as the linker residues between domains (160-200) [17].

Considered a multifunctional enzyme due to its involvement in various biological processes, CatD operates in both cytosolic and extracellular environments [13, 18-22]. Studies have shown that CatD is involved in the activation of precursors of biologically active proteins in pre-lysosomal compartments of specialized cells [11, 20, 23]. This enzyme is indispensable for cellular functions such as cell migration, differentiation, growth, cycle progression, tissue remodeling, and neovascularization activation [6, 11, 12, 19, 22, 24-27]. Additionally, CatD is involved in initiating the apoptotic cascade [28, 29] in lysosomal cell death pathways [22, 25].

CatD is directly related to the pathogenesis and progression of degenerative diseases [6, 30], such as lymphoid cell degeneration [31], Parkinson’s [32] and Alzheimer’s disease [33], atherosclerosis [34], and different types of cancer [35, 36]. For instance, some cell types under pathological conditions overexpress and secrete CatD to the extracellular environment via lysosomal release [20]; this makes CatD an important tumor marker in breast, bladder, and mouth cancers, among others [35, 36]. Furthermore, due to the participation of cathepsins in a broad spectrum of diseases, these proteases are promising therapeutic targets for small molecules and peptide drugs [33, 36].

In order to investigate human CatD inhibitors for the design and development of tools and agents of scientific and therapeutic interest, snake venoms belonging to the genera *Bothrops*, *Crotalus*, and *Lachesis* have been used as natural sources of biologically active molecules able to act selectively and specifically on different cellular targets [37, 38]. Of all the bioactive molecules present in snake venoms, phospholipases A_2_ (svPLA_2_) are among the most frequently encountered and studied [39, 40]; these proteins have established physical-chemical properties and a variety of pharmacologic and toxic effects in snakebite envenomation, such as myonecrosis, anticoagulation, platelet aggregation inhibition, neurotoxicity, cardiotoxicity, hypotension and edema formation [41-45].

Interestingly, human CatD shows the ability to bind strongly to svPLA_2_s, forming a stable and functional complex that is able to remain active even at pH values higher than 5 and is also unaffected by the inhibitor pepstatin A. These results, presented and discussed below, demonstrate the multifunctionality and versatility of CatD, warranting many new possibilities for the understanding of cathepsin functions in cytosolic and extracellular environments during physiologic and pathologic processes. Therefore, the present study aims to demonstrate and characterize an enzymatic complex formed by human CatD and a snake venom phospholipase A_2_.

## Methods

### Cathepsin D

Cathepsin D (cod. C8696) was obtained from Sigma-Aldrich Ltda and prepared according to the manufacturer’s recommendations.

### Snake venoms

All snake venoms used in this study were acquired from the Venom Bank at CEBio/Fiocruz Rondônia/UNIR (Centro de Estudos de Biomoléculas Aplicadas a Saúde), Porto Velho, RO, under local government authorization license number: IBAMA nº 27131-3 and CGEN/CNPq 010627/2011-1.

### Phospholipases A_2_ (PLA_2_s)

The Bothropstoxin-I (BthTX-I) and Bothropstoxin-II (BthTX-II) from *Bothrops jararacussu* were obtained from the Venom Bank at CEBio (Centro de Estudos de Biomoléculas Aplicadas a Saúde/Fiocruz Rondônia/UNIR), located in Porto Velho, RO. PLA_2_ LmtTX from *Lachesis muta* provided by Diniz-Sousa et al. [46], PLA_2_ BnuTX-I from *Bothrops urutu* provided by Corrêa et al. [47], PLA_2_ Braziliase-I and Braziliase-II from *Bothrops brazili* provided by Kayano et al. [48] and Sobrinho et al. [49]. 

### *Bothrops jararaca* snake venom fractionation

*B. jararaca* venom was solubilized in 50mM ammonium bicarbonate buffer (AMBIC), pH 8.0 and applied to an anion exchange column (CM-Sepharose 10 x 30 cm). The fractions were eluted in a linear gradient of 500 mM AMBIC, pH 8.0 under a flow of 1 mL/min. Absorbances were measured at 215 and 280 nm. The fractions were subjected to salt removal in a 15mL filter (AMICON ULTRA-15) with a 50 kDa cutoff.

### Binding assays

Surface plasmon resonance (SPR) molecular interaction assays were performed in a Biacore T200 system (GE Healthcare). Cathepsin D immobilization was done using a CM5 S-type sensor chip via amine coupling. The contact time of each cycle was set at 60 seconds, with a flow rate of 30 µL/min, followed by 60 seconds of dissociation time. For the regeneration stage at the end of each cycle, a 0.5% TFA solution was used with 30 seconds of contact time at a flow of 30 µL/min. All experiments were performed at 25 ºC, and binding assays were conducted in phosphate-saline buffer (PBS), pH 7.4 and analytes at a concentration of 100 µg/mL.

### Protein quantification

The protein concentrations present in venom samples were determined using Bradford’s method [50]. For spectrophotometric measurements, the sample was aliquoted in a 1 mL disposable plastic cuvette along with 1:10 (v/v) Bradford reagent, which was incubated for 15 minutes. Absorbance was monitored at 595 nm using a Biomate 3 spectrophotometer. The calibration curve was performed using bovine albumin (Sigma).

### SDS-PAGE

The relative mass of proteins was determined by SDS-PAGE using discontinuous gels, with a stacking gel (4% acrylamide in 0.5 M Tris-HCl buffer, pH 6.8) (Sigma Aldrich, USA) and a resolving gel (12.5% acrylamide in 1.5 M Tris-HCl buffer, pH 8.8). The experimental buffer solution used to fill the wells was 0.06 M Tris-Base, 0.5 M Glycine, and 10% SDS (Sigma Aldrich, USA). The samples with 1M DTT were preheated to 95 °C for 5 min and applied to the stacking gel wells along with the Molecular Weight standard (7 to 175 kDa - BioLabs P7709S, USA). In the electrophoretic run, a current of 15 mA per gel and free voltage was fixed for 1 hour and 40 minutes. After this, the gel was washed for 15 minutes with a fixing solution (ethyl alcohol 50% and acetic acid 12%) and then stained with Coomassie G-250 blue solution (Sigma Aldrich, USA) for 10-30 minutes. After this period, the gel was bleached in a bleaching solution (20% ethyl alcohol and 3% acetic acid). The gels’ images were scanned using Image Scanner III (GE Lifescience Health Care).

The 2D electrophoresis consisted of two steps: isoelectric focusing and 1D SDS-PAGE. For the first dimension, the sample was prepared in a rehydration solution (8 M urea, 2% CHAPS, 0.5/2% IPG buffer, 0.002% bromophenol blue, and 1 M DTT); this same solution was then incubated with a 7-cm strip (pH 3-10, linear) for 12-20 h. After rehydration, the strip was applied to an Ettan IPGphor 3 (GE Healthcare) isoelectric focusing system and later stored at − 80 °C. For the second dimension, the strip was washed with DTT and iodoacetamide diluted in 5 mL of equilibration buffer solution (6 M urea, 2% SDS, 30% glycerol, 50 mM Tris- HCl, pH 7.4, 0.002% bromophenol blue). Then, the strip was applied to a 15% polyacrylamide gel. The gel was stained with Coomassie Blue G-250 and scanned in a GE Image Scanner III apparatus.

### Metalloprotease contamination analysis of BthTX-II

Proteolytic activity was evaluated according to the method described by Rodrigues and coworkers [51], with adaptations, using casein as a substrate. Samples (12 µg/mL) were incubated with 250 µL of 2% casein in 0.1 M sodium citrate (pH 3, 4, 5, 6, 7) for 30 minutes at 37 °C, interrupted by the addition of 250 μL of 20% trichloroacetic acid (TCA). Similarly, sample contamination by metalloprotease at different pHs was analyzed by adding 10uL of ethylenediaminetetraacetic acid (EDTA). The solution was left to rest for 30 minutes at room temperature and then centrifuged at 10,000 x g for 15 minutes at 25 °C. The proteolytic activity was estimated based on the absorbance of the supernatant at 280 nm, with trypsin as a positive control.

### Proteolytic activity on casein

The proteolytic activity was evaluated according to the method described by Rodrigues et al. [51], with adaptations, using casein as a substrate. Samples (6.3 µg/mL) were incubated with 250 µL of 2% casein in 0.1 M sodium citrate (pH 3, 4, 5, 6, 7) for 30 minutes at 37 °C and then interrupted by the addition of 250 µL of 20% trichloroacetic acid (TCA). The solution was left to stand for 30 minutes at room temperature and then centrifuged at 10,000 x g for 15 minutes at 25 °C. Proteolytic activity was estimated based on the absorbance of the supernatant at 280 nm. The proteolytic activity monitored in SDS-PAGE electrophoresis was performed according to the protocol described above. Inhibition was carried out by means of exposure to high temperatures (90 ºC).

### Phospholipasic activity on 4N3OBA

This procedure was carried out as described by Petrovic and coworkers [52]. 5 mg of the substrate 4-nitro-3-octanoyloxy-benzoic acid (4N3OBA) (Enzo Lifescience, USA) was diluted in 5.4 mL of acetonitrile. 0.2 mL aliquots were dried and stored at -20 °C. Each tube containing 4N3OBA was diluted in 2 mL of sample buffer (0.01 M Tris-HCl at pH 8.0, 0.01 M CaCl_2_, and 0.1 M NaCl) (Sigma Aldrich, USA) and maintained on ice. In order to determine the phospholipasic activity, a total of 190 μL of 4N3OBA reagent combined with 10 μL of sample (cathepsin + BthTX-II, and inhibitor) was applied in a 1:1 ratio, pre-diluted in water and incubated at 37 °C; subsequently, the substrate was added to the samples and immediately incubated at 37 ºC. The absorbance was measured at 425 nm for 30 minutes (interval of 1 min). Phospholipase activity was considered directly proportional to the increase in absorbance values and expressed as the mean ± standard deviation; the results were submitted to analysis of variance (ANOVA) followed by Tukey’s post-test for p < 0.05.

### *In silico* molecular interactions

All PLA_2_s used in the *in vitro* assays were assessed through molecular docking against cathepsin D (CatD). The available structures of CatD (4OD9), BthTX-I (3CXI), BthTX-II (2OQD), and Crotoxin B (3R0l) were extracted from the RCSB Protein Data Bank. The structures of Braziliase II (UniProtKB: P0DUN4) and LmutTX (UniProtKB: P0DUN7) were generated by means of comparative modeling using the Rosetta web server [53]. The structural conformation guiding the interaction and complexation of CatD and PLA_2_ were predicted through a consensus of 5 protein/protein docking tools (pyDock, ZDOCK, HDOCK, ClusPro, and GRAMX). The CatD + BthTX-II complex was subjected to molecular dynamics, with five replicas of 100 ns using GROMACS 2020.2 employing the CHARMM36-mar2019 force field [54]. All simulations were carried out with a neutral net charge box of 4 Å radius from the farthest atom, solvated with TIP3P water, and equilibrated with 100 mM NaCl. The system was minimized with the steeper descent minimization until it reaches the power levels below 100 kJ/mol/nm. 

Then, the box was equilibrated under an isochoric-isothermal (NVT) ensemble for 1 ns, generating speeds according to the distribution of Maxwell-Boltzmann at 310.15 K using the V-Rescale thermostat [55] followed by an isothermal-isobaric (NPT) ensemble using the Berendsen barostat at 1 bar [56]. Subsequently, five replicas of unrestrained 100 ns simulations were executed using the Nose-Hoover Thermostat [57] and Parrinello-Rahman barostat [58].

Nonbonded interactions were calculated within a radius of 12 Å using a switching function between 10 and 12 Å. Afterwards, the trajectories were analyzed, and radius of gyration and backbone RMSD measurements were extracted from the main interacting parties for stability assessment. Further, the trajectories were subjected to clusterization using the gromos method [59] with an RMSD distribution of 2 Å. All images and interaction maps were created using UCSF Chimera 1.13.1 [60].

## Results

### Snake venom binding assays

**Figure 1.**
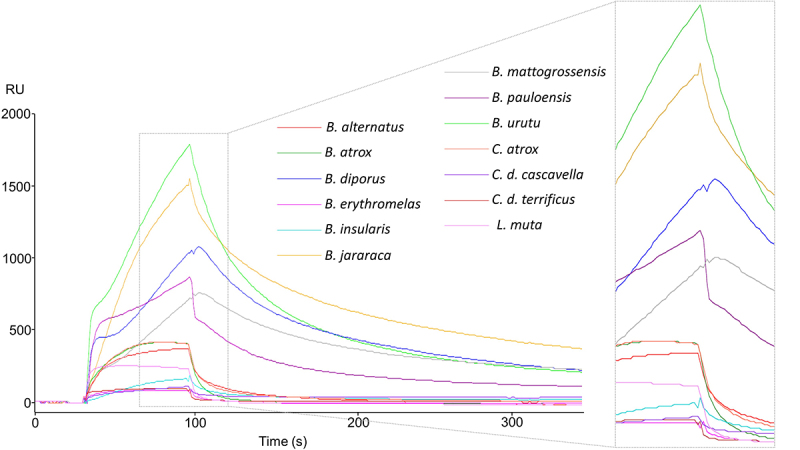
SPR assays between snake venoms and human cathepsin D (CatD). Sensorgrams were generated in a screening round of snake venoms against CatD. All interactions are plotted according to the response upon binding in RU (resonance units).

Snake venoms were screened as to their potential interactions with human CatD, aiming to generate an extensive analysis of binding responses featuring the unique molecular content found in each venom. In this fashion, the bioactive compounds with the most affinity towards CatD could be inferred based on venom composition. For this purpose, thirteen venoms from different species were used (Fig. 1 and Table 1). Among these species, *Bothrops brazili, B. jararaca, B. jararacussu,* and *B. leucurus* stood out as promising due to their association and dissociation profiles and the maximum number of responses reached. 

**Table 1.** SPR binding assay of immobilized human cathepsin D with crude snake venoms.

Species	Protein concentration (mg/mL)^a^	Response (RU)^b^	RU/mg
*Bothrops alternatus*	1.096	342.0	312.0
*B. atrox*	0.954	378.6	396.9
*B. brazili*	1.023	1,549	1,514.2
*B. diporus*	1.051	973.8	927.0
*B. erythromelas*	1.147	76.5	66.7
*B. insularis*	0.544	134.0	246.0
*B. jararaca*	0.867	1,409.3	1,625.5
*B. jararacussu*	0.887	2,003.1	2,258.3
*B. leucurus*	0.965	1,508.0	1,562.7
*B. mattogrossensis*	1.122	665.6	593.2
*B. pauloensis*	0.734	804.3	1,095.8
*B. urutu*	1.457	1,663.3	1,141.6
*Crotalus atrox*	1.470	185.0	125.9
*C. d. cascavella*	2.934	100.0	34.1
*C.d. terrificus*	0.779	65.0	83.4
*Lachesis muta*	1.689	216.7	128.3

^a^ Protein quantification using the Bradford Method. ^b^Response: maximum response values are presented in resonance unit (RU).

**Figure 2.**
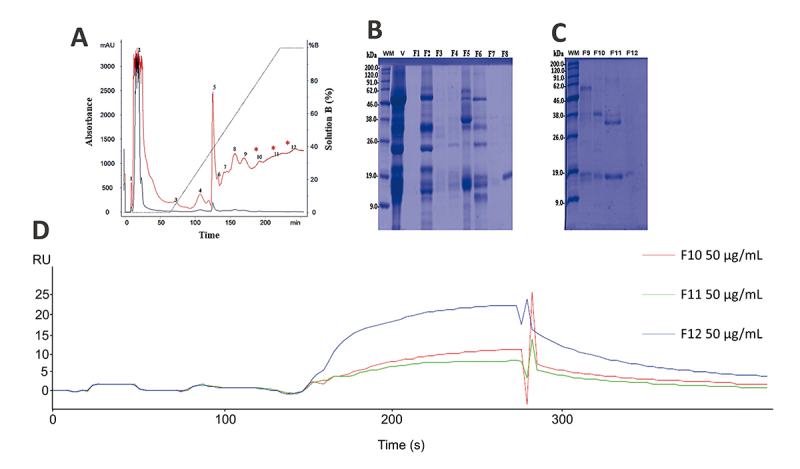
Chromatographic profile of *B. jararaca* snake venom, SDS-PAGE, and binding assays of the isolated fractions. **(A)** The chromatographic profile demonstrates fractionation on a CM-Sepharose column previously equilibrated with 50 mM AMBIC, pH 8.0, and fractions eluted with a 0-100% gradient of 500 mM AMBIC, pH 8.0, at a constant flow rate of 1 mL/min, monitored at 215 (red) and 280 nm (blue). The twelve fractions collected were numbered from 1 to 12, the fractions of interest 10, 11, and 12 being indicated with asterisks (*). SDS-PAGE of the 12 fractions from *B. jararaca* venom. **(B)** MM: molecular mass, V: crude venom, and eight fractions named F1 to F8. **(C)** MM: molecular mass, and fractions from F9 to F12. **(D)** Fraction interaction responses: 10 (blue), 11 (green) and 12 (red) with responses of 125, 10 and 12 RUs, respectively.

*Bothrops jararaca* venom is one of the most well-characterized and studied venoms and showed a significant binding response (1,625 RU mg/mL) with CatD; for those reasons, it was selected for further analysis. In order to identify the venom components responsible for the majority of interaction signals, *B. jararaca* crude venom was fractionated through cation exchange chromatography (Fig. 2A). The chromatography resulted in 12 fractions that were later submitted to SPR assays against CatD.

The subsequent assays revealed that only fractions 10, 11, and 12 presented significant interactions with CatD, showing responses from 25, 12, and 10 RUs at a concentration of 50 mM (Fig. 2D). Next, the protein profile of each fraction was determined by SDS-PAGE, resulting in clear monophoretic bands around 13 kDa for all three fractions (Fig. 2C), compatible with svPLA_2_ mass and bands between 30 to 40 kDa, suggesting snake venom metalloproteases (SVMPs) in the fractions 10 and 11 (Fig. 2C). When these fractions were tested for their phospholipase activity, fractions 3, 8, 10, 11, and 12 showed relevant activity against the substrate 4N3OBA (results not shown), confirming the presence of phospholipases in the fractions of interest.

These data strongly suggested that human CatD has the ability to interact with svPLA_2_s. In order to investigate this tendency and evaluate the specific affinity between both proteins, six svPLA_2_s from the genera *Bothrops* and *Lachesis* were submitted to SPR assays at concentrations of 15 and 50 mM (Table 2).

**Table 2.** SPR interaction assays of immobilized human cathepsin D with svPLA_2_s from *Bothrops* and *Lachesis* snake species.

svPLA_2_	Type	Species	Concentration (mM)	Response (RU)
BthTX-I	Lys-49	*B. jararacussu*	50/15	NC
BthTX-II	Asp-49	*B. jararacussu*	50/15	1,420/420
Braziliase-I	Asp-49	*B. brazili*	50/15	NC
Braziliase-II	Asp-49	*B. brazili*	50/15	837/245
BnuTX-I	Lys-49	*B. urutu*	50	552
LmutTX	Asp-49	*L. muta*	50	2,180

Samples that showed distorted results were considered inconclusive (NC).

**Figure 3.**
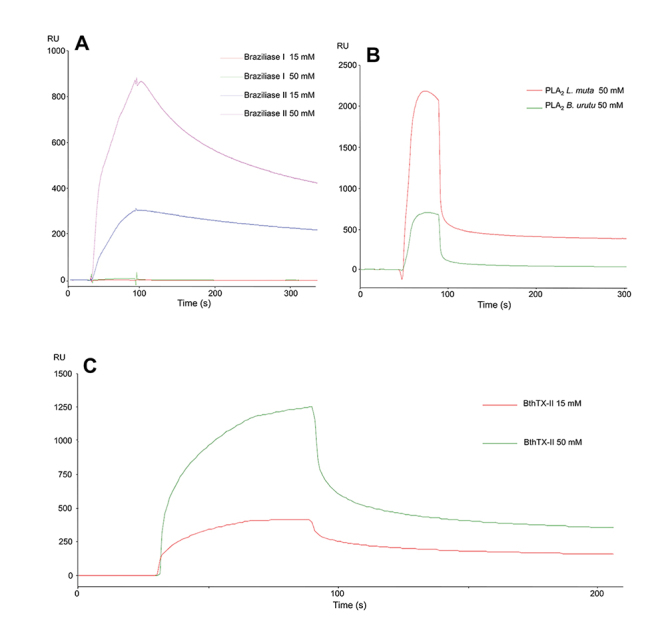
Binding assays between CatD and snake venom PLA_2_s. **(A)** Interactions of CatD with Braziliase-I and Braziliase-II (concentrations of 15 and 50 µM). **(B)** Responses were obtained from the interaction between CatD and svPLA_2_s from *Bothrops neuwiedi urutu* (BnuTX-I) and *Lachesis muta* (LmutTX). **(C)** Interaction test between CatD and BthTX-II (concentrations of 15 and 50 mM). The analyzed samples were submitted to salt removal in a 5 mL Hitrap desalting (GE) column.

The binding analysis via SPR spectroscopy revealed that the toxins tested (except BthTX-I and Braziliase I) displayed tight binding to immobilized CatD (Fig. 3). For instance, BthTX-II (an enzymatic Asp-49-PLA_2_) [61] presented interaction showing dose-dependent SPR responses ranging from 420 to 1,420 at concentrations of 15 and 50 mM, respectively (Fig. 3C).

Different from Braziliase-I, Braziliase-II showed a dose-dependent sensorgram of 245 RUs (15 mM) and 837 RUs (50 mM) with a prolonged dissociation phase suggesting a possible low dissociation rate constant (Kd) (Fig. 3A), which could be investigated through further analysis. Both BnuTX-I from *B. urutu* and LmutTX from *L. muta* also interacted with immobilized CatD (Fig. 3B), showing sensorgrams with different intensities of 552 and 2,180 RU at 50 mM [46, 47]. In any case, both showed a similar shape in their association and dissociation curves.

Despite the high level of homology among svPLA_2_s, the binding analysis between CatD and these toxins exhibited interactions with different intensity profiles. Nevertheless, the binding profile of CatD towards svPLA_2_ displayed high similarity, suggesting a common recognition site. It is worth pointing out that overall, svPLA_2_s present a characteristic and consistent tridimensional structure, which could be the driving factor behind the ability of CatD to interact with the svPLA_2_s tested in this study [61, 62].

### Enzymatic activity of the cathepsin D + BthTX-II complex

**Figure 4.**
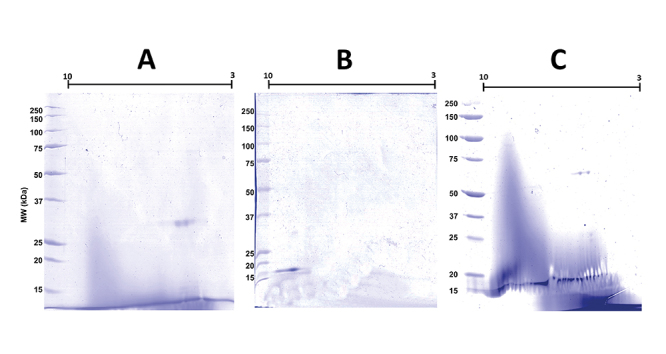
Two-dimensional SDS-PAGE: CatD, BthTX-II, and enzymatic complex. **(A)** Two-dimensional SDS-PAGE of CatD showing a pI of 4.74 and approximate molecular mass of 35 kDa. **(B)** Two-dimensional SDS-PAGE of BthTX-ll with a pI of 8.74, with an approximate molecular mass of 14 kDa. **(C)** Two-dimensional SDS-PAGE of the complex with a pI of 5.79, and approximate molecular mass of 49 kDa.

**Figure 5.**
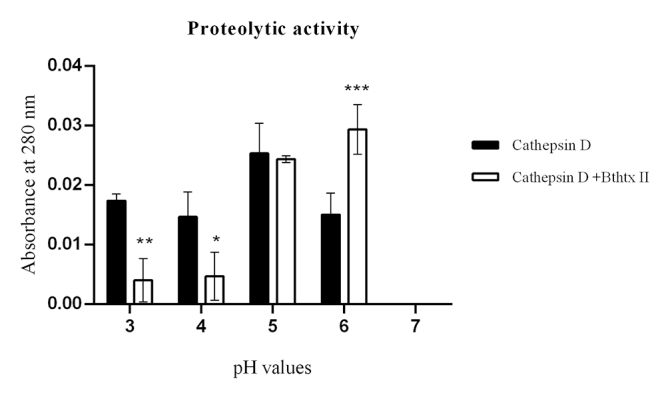
Proteolytic activity of CatD and the CatD + BthTX-II complex. The evaluation was performed at pHs 3, 4, 5, 6, and 7, identified in the figure legend, highlighting CatD in white (positive control) and the CatD + BthTX-II complex in black. As a negative control, the buffer itself (sodium citrate) was used at different pHs. The toxin used in the tests (BthTX-II) was submitted to contamination analysis (described in the second section). Two-way analysis of variance (ANOVA) with Tukey’s multiple comparison post-test with significance level p < 0.05.

**Figure 6.**
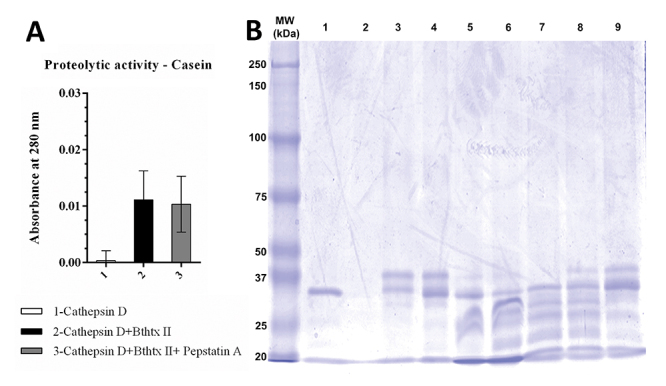
Proteolytic activity on casein. **(A)** Samples identified in the legend results: CatD, CatD + BthTX-II, CatD + BthTX-II + pepstatin A were considered negative controls; buffer (sodium citrate) was used at different pHs. **(B)** Proteolytic activities SDS-PAGE. Samples: (1) CatD; (2) pepstatin A (PepA); (3) casein; (4) CatD + PepA + casein; (5) CatD + casein; (6) CatD + BthTX-II Casein + PepA; (7) CatD + BthTX-II + casein (30 min); (8) CatD + BthTX-II + casein (15 min); (9) CatD + BthTX-II + casein (5 min). Samples 3 through 6 were incubated for 30 min at 27 ºC. Positive control: CatD; negative control: pepstatin A (PepA).

Initially, the apparent molecular mass and isoelectric point (pI) of the CatD + BthTX-II complex, as well as that of both enzymes separately, BthTX-II and CatD, were verified through two-dimensional electrophoresis (Fig. 4), determining a molecular mass of approximately 60 kDa and pI of 5.79 for the CatD + BthTX-II complex. Next, the proteolytic activity of CatD and of its complex with svPLA_2_ (BthTX-II) were evaluated using casein as a substrate at pH values of 3, 4, 5, 6, and 7, and Pepstatin A as a specific inhibitor. The optimal enzymatic activity of CatD was observed at pH 5, which is in agreement with previous studies [63]. On the other hand, the CatD + BthTX-II complex proved to be functional at different pH values reaching maximum activity at pH 6 (Fig. 5), revealing that the binding between these two proteins changes CatD’s functionalities, increasing its pH-dependent activity to higher values. Additionally, the CatD + BthTX-II complex is resistant to the inhibitor Pepstatin A at pH 6, suggesting the possibility of changes in enzyme specificity (Fig. 6A). 

Similar outcomes were observed in the SDS-PAGE assay, revealing that the bands formed after casein hydrolysis by CatD and CatD + BthTX-II are slightly different (Fig. 6B), suggesting potential differences in cleavage sites and further confirming the *in vitro* enzymatic activity. Furthermore, to rule out any residual contamination from the BthTX-II sample due to venom proteases, this sample was also submitted to the same conditions, and showed no proteolytic activity (results not shown).

**Figure 7.**
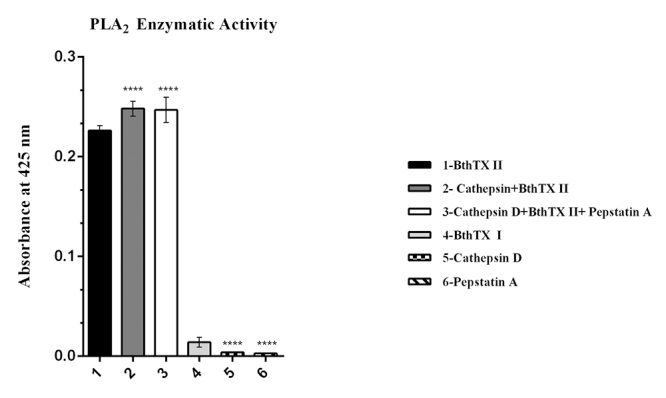
PLA_2_ enzymatic activity on artificial substrate 4N3OBA. Samples: (1) BthTX-II; (2) CatD + BthTX-II; (3) CatD + BthTX-II + PepA; (4) BthTX-I; (5) CatD; (6) PepA. Positive control: BthTX-II. Negative control: BthTX-I.

Regarding the effects of the interaction of the CatD + BthTX-II complex on BthTX-II’s catalytic function, the phospholipase activity assay (Fig. 7) shows that the complex’s formation does not interfere with nor hinder BthTX-II’s capability to cleave the artificial substrate 4N30BA. Interestingly, the presence of Pepstatin A slightly diminishes the catalytic output of the CatD + BthTX-II complex.

### Structural analysis and molecular interaction simulations

**Figure 8.**
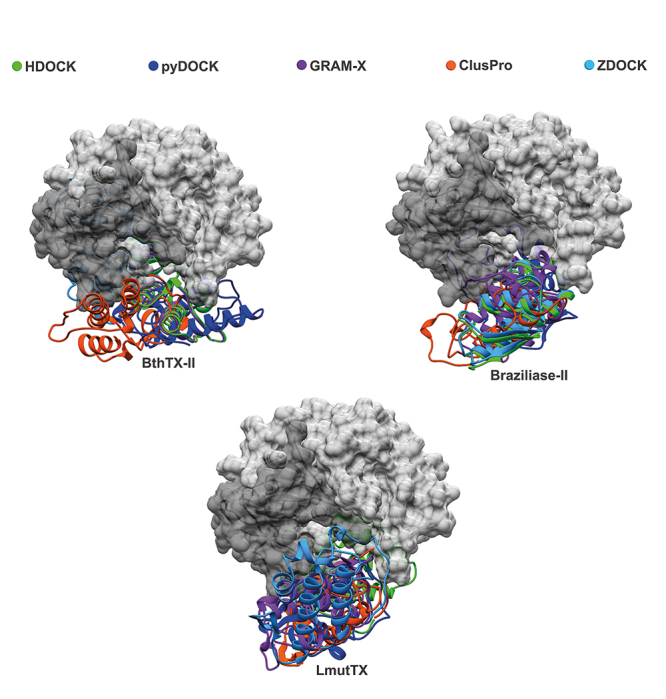
Molecular modeling of the interaction between three snake venom PLA_2_s (LmuTX, Braziliase-II and BthTX-II) and human CatD using different docking tools (HDOCK, pyDOCK, GRAM-X, ClusPro, and ZDOCK). The CatD surface is represented in dark gray (light chain) and light gray (heavy chain). The svPLA_2_s are colored according to the docking tool used.

**Figure 9.**
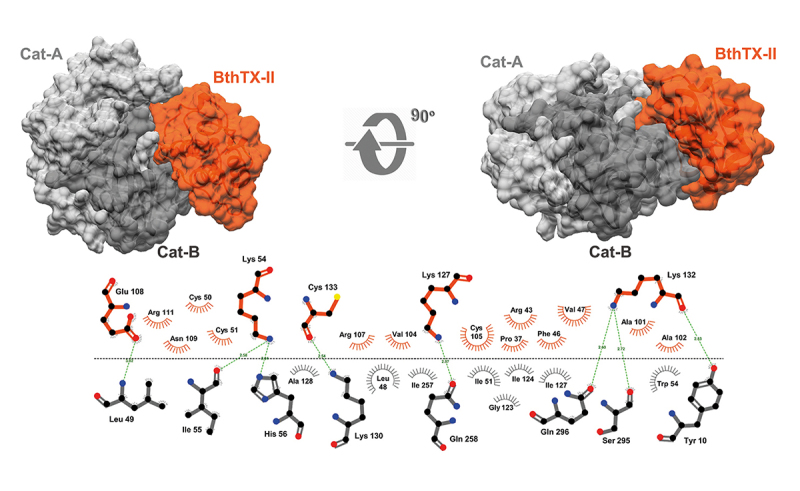
Molecular modeling of the CatD + BthTX-II complex. The complex formed between human CatD is shown in gray (light chain in dark gray and heavy chain in light gray) and BthTX-II is shown in orange. The interactions were enlarged to show amino acid residues in the interface and their interactions. H-bonds are highlighted by green dashed lines, and hydrophobic interactions are depicted as protrusions colored to match each amino acid residue.

**Figure 10.**
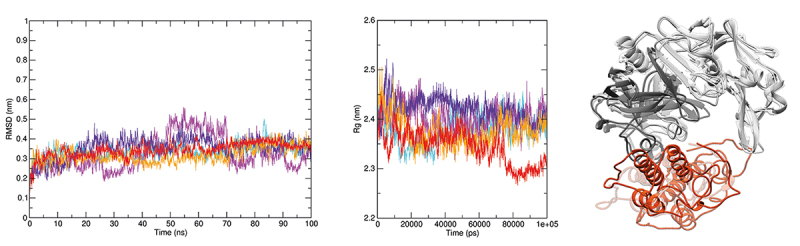
CatD + BthTX-II complex molecular dynamics. CatD is shown in gray (light chain in dark gray and heavy chain in light gray) and BthTX-II is shown in orange. The radius of gyration and backbone RMSD graphics are located on the left side of the figure. All five replicas are plotted in an overlapped manner in order to highlight all minor variations and overall stability throughout the 100 ns of each replica. The structures on the right end of the figure show a superposition respective to each of the three CatD + BthTX-II complexes representing the most predominant conformations during the total 500 ns simulated. These superposed complexes are the central structures extracted from the three most populated clusters generated in the clusterization analysis performed with the trajectories of all five replicas.

All svPLA_2_s showing interaction with CatD in the SPR assay and enzymatic assays were selected for further *in silico* investigation, seeking details about the mechanism coordinating these interactions at the atomic level and the existence of common recognition sites for svPLA_2_s on CatD’s surface. Thus, five molecular docking methodologies were applied, effectively employing a consensus approach, which generated sets of docking conformations (Fig. 8) for each of the svPLAs2 (BthTX-II, Braziliase-II and LmutTX). Additionally, the CatD + BthTX-II complex (Fig. 9) was subjected to a more intensive inspection due to its enzymatic activity. Molecular dynamics (MD) was used to evaluate the structural stability of this macromolecular assembly. Five independent replicas were simulated for 100 ns each. The processing and analysis of the generated trajectories included an assessment of the CatD + BthTX-II complex’s behavior in solution considering the radius of gyration (Fig. 10A) and RMSD (Fig. 10B) variations during the simulations. There were few noticeable fluctuations in the complex’s backbone and its compactness. Nevertheless, the assembly formed between these two proteins remained stable through all five replicas. The interaction between CatD and BthTX-II was evaluated, using as reference the central structures from the three most populated clusters generated in the clusterization performed with the sum of all five trajectories, exhibiting in that way an approximation of the most predominant conformation assumed by the CatD + BthTX-II complex during 500 ns of simulation (Fig. 10C). The absence of any remarkable shift in the complex’s shape suggests an overall stable and cohesive interaction. 

## Discussion

In order to proceed with the characterization of the CatD + BthTX-II complex, different methodologies were used, such as Surface Plasmon Resonance (SPR), a detection method capable of performing real-time, label-free, and high-sensitivity monitoring of molecular interactions [64], and molecular docking, a key tool in structural molecular biology and computer-aided drug design, useful to predict structural data about a potential protein-protein interaction using known three-dimensional structures [65].

SPR assays carried out with immobilized human CatD showed different levels of interaction with components of all snake venoms tested, ranging from 34.1 RU mg/mL for *C. d. cascavella* to 2,258.3 RU mg/mL for *B. jararacussu* (Table 1). The interaction of venom components with human cathepsin D, especially those from bothropic venoms, strongly suggests that this could be an important and relevant new biological mechanism involving the participation of CatD and svPLA_2_ in snake envenomation and other physiopathological processes with the participation of homologous proteins.

The use of *B. jararaca* venom cation exchange chromatographic fractions for further SPR assays (Fig. 2) showed that immobilized CatD interacted only with the last fractions (10, 11, and 12), which corresponds to well-known svPLA_2_s, according to the monophoretic bands observed in the electrophoresis profile. This data indicated that the svPLA_2_s presented in the samples tested in SPR binding assays with CatD could be the respective ligands. The SPR analyses carried out with the isolated svPLA_2_s BthTX-II, Braziliase-II, BnuTX-I, and LmutTX revealed their ability to bind with immobilized human CatD (Fig. 3). 

Two-dimensional electrophoresis showed that human CatD and BthTX-II form a stable complex of approximately 60 kDa and pI of 5.79. Initially, the apparent molecular mass and isoelectric point (pI) of the CatD + BthTX-II complex, as well as that of both enzymes separately, BthTX-II and CatD, were verified through two-dimensional electrophoresis (Fig. 4), determining a molecular mass for the CatD + BthTX-II complex. Next, the proteolytic activity of CatD and its complex with svPLA_2_ (BthTX-II) was evaluated using casein as a substrate at pH values from 3 to 7, and Pepstatin A as a specific inhibitor. The pH optimum of the CatD + BthTX-II complex was found to be 6, while isolated CatD shows optimal activity at pH 4 [66]. Furthermore, Pepstatin A doesn’t affect the CatD + BthTX-II complex activity with the substrate (Casein) at different pH values. 

Interestingly, the change in CatD pH-dependent activity, when compared to that of the CatD + BthTX-II complex, is consistent with previous CatD studies in tumoral cell lines [67], suggesting that in the physiologic scenario, CatD’s interaction with proteins such as svPLA_2_ might be the factor allowing it to function in different pH ranges. Additionally, the CatD + BthTX-II complex was not inhibited by Pepstatin A, with CatD’s catalytic activity remaining steady, further corroborating the CatD + BthTX-II complex’s increased activity capacity. Moreover, the investigation of the CatD + BthTX-II complex’s impact on BthTX-II’s phospholipase activity suggests that the orientation of BthTX-II when coupled with CatD is ideal and allows BthTX-II to remain fully functional.

Computational simulations revealed a clear pattern of interaction between CatD and svPLA_2_s, in such a way that all svPLA_2_s tested in this study exhibited affinity by the concave surface formed between the heavy and light chain of CatD. This interaction profile was observed in every docking performed in this study. Furthermore, MD simulations done with the CatD + BthTX-II complex demonstrated that this may be the stable conformation assumed by CatD interacting with svPLA_2_s in solution. Alone, the CatD + svPLA_2_ complex’s interface of interaction observed in the simulations performed herein is not able to enlighten the molecular mechanisms behind the boost in CatD’s catalytic activity observed in the enzymatic assays. However, the conformation of the CatD + BthTX-II complex generated in the docking predictions and later validated in the 500 ns of simulations agrees with the phospholipase activity assays. The capability of the CatD + BthTX-II complex to retain svPLA_2_ makes perfect sense given BthTX-II’s orientation upon attachment to CatD (Fig. 9 and 10C), in such a way that BthTX-II’s hydrophobic channel and active site remain fully exposed to solvent.

Taking into account all these data, the *in silico* exploration of CatD’s complex with svPLA_2_ provides a clear basis for these two enzymes’ interaction in the physiologic scenario. Nevertheless, it is necessary to carry out more experimental structural studies in order to confirm the modes of interaction between these enzymes. These results also raise new questions in the investigation of pathological and inflammatory symptoms of snake envenomation, in which CatD’s interaction with svPLA_2_ and the complexes formed could play an important role in the cascade of systemic and local effects present in snakebite accidents. 

The interaction between CatD and svPLA_2_ demonstrated herein will possibly have future implications for snakebite therapeutics. However, the most significant results extracted from this study may foreshadow more fundamental physiological issues involving the role of CatD in inflammatory processes, apoptosis and tumor progression. In this regard, the proteolytic process in neurons, in which CatD actively participates, is an essential maintenance step for the clearance of protein aggregates that reach the lysosomes through endocytosis and autophagy [24]. 

Di Domenico and coworkers proposed that the lack of control in protein repair (proteasome and lysosomal system) is a characteristic of degenerating neurons in Alzheimer’s disease (AD), which highlights CatD’s involvement in these conditions due to its essential role in the management of lysosomal integrity [33]. Thus, the rise in PLA_2_ (IIA) in the cerebrospinal fluid of patients with AD indicates these enzymes as potential biomarkers in neuroinflammation [68, 69]. Furthermore, human brains affected by AD present a significant increase in PLA_2_ mRNA in the hippocampus [70]. Interestingly, reports of PLA_2_s’ involvement in the destabilization of lysosomal membranes have been made in different experimental systems [29, 71, 72]. 

Overall, many approaches have discussed the involvement of PLA_2_s in inflammatory processes [73-75]. In addition, PLA_2_s also act on cell membrane metabolism and the production of arachidonic acid, a known precursor of prostaglandins, leukotrienes, and thromboxanes [76-78]. Johansson and coworkers demonstrated that incubation of PLA_2_s with rat liver lysosomes resulted in the extravasation of its lysosomal constituents [29]. Additionally, Beaujouin and coworkers demonstrated CatD’s involvement in apoptosis and showed that cancer cells that were pretreated with Pepstatin A, could not halt CatD nor hinder apoptosis, supporting the results described herein in the proteolytic activity assays. Moreover, CatD’s capability to induce cancer cell growth, even when mutated, suggests an alternative mechanism for this enzyme [79].

## Conclusion

For the first time, this study describes the formation of a functional muti-enzymatic complex between the human protease cathepsin D and snake venom phospholipases A_2_. Collectively, the *in vitro* assays and *in silico* predictions carried out in this study demonstrated interaction and the formation of a new muti-enzymatic and catalytically active complex between CatD and svPLA_2_. Additionally, the agreement between the data from previous studies regarding the pathways in which these enzymes are involved and the new data presented herein indicates the possibility of PLA_2_ and CatD acting in conjunction in the extracellular environment [41]. Nevertheless, in the face of the many possible outcomes of this new enzymatic complex, the conclusions drawn must be taken with caution and, most importantly, warrant more extensive investigation on the subject.
